# In memoriam: Kuan-Teh Jeang, MD PhD (1958–2013)

**DOI:** 10.1186/2045-3701-3-13

**Published:** 2013-02-28

**Authors:** Dong-Yan Jin, Yun-Bo Shi, T-C Wu

**Affiliations:** 1 Department of Biochemistry, The University of Hong Kong, Pokfulam, Hong Kong; 2 The National Institutes of Health, Bethesda, Maryland, USA; 3 Department of Pathology, Johns Hopkins University, Baltimore, Maryland, USA

## 

Talking about the golden era of the National Institutes of Health (NIH), Nobel laureates Joseph Goldstein and Michael Brown concluded in a recent issue of *Science* that "ambitious young physicians juxtaposed to cutting-edge basic scientists can themselves make fundamental discoveries" [1]. In our minds, both the late George Khoury and his postdoc Kuan-Teh Jeang, known as “Teh” among friends and colleagues, were in that group of brilliant NIHers who broke new ground in many areas of biomedical research, particularly in the study of retroviruses. When one of us, Dong-Yan Jin, joined Teh's laboratory at NIH in 1994, he learned from Teh not only about George Khoury’s scientific achievements but also about his strong commitments to friends, family and community. Although he died much too early at the age of 43 in 1987, George was Teh's role model and influenced him greatly in his professional life. They shared many things in common, most importantly their devotion to science and people. As a recognition of his scientific achievements, Teh was chosen to deliver the 2012 George Khoury Lecture at NIH in October 2012 on "Nuclear damage and aneuploidy: human T cell leukemia virus transformation of cells". Teh was such an energetic and tireless person that NIH deputy Director Michael Gottesman described him as a dynamo at that lecture. Teh's sudden and unexpected passing in the late evening of January 27, 2012 saddened all of us completely.

Teh had been working at the NIH for 27 years and was chief of the Molecular Virology Section in the Laboratory of Molecular Microbiology of the National Institute of Allergy and Infectious Diseases. He led a highly productive group and published more than 250 scientific papers in the broad field of biomedical science, with a focus on two human retroviruses: human immunodeficiency virus type 1 (HIV-1) and human T-cell leukemia virus type 1 (HTLV-1). Many of his scientific discoveries were seminal and paradigm-shifting. His findings revealed new clues to fundamental questions such as how HIV-1 transcribes its RNA and how HTLV-1 transforms cells. They had important implications in disease prevention and intervention. Teh trained more than 30 postdocs, many of whom moved on to faculty positions in the US, Europe and Asia. Their association with Teh was critical to the advancement of their scientific career.

Teh was a scientific leader in HIV research. In 1989 Teh discovered that the activation of HIV-1 long terminal repeats by viral transactivator Tat requires a highly structured RNA hairpin termed TAR [2]. This was a conceptual breakthrough that forever changed the field of transcriptional regulation. In addition, it also provided the foundation for rational design and development of RNA therapeutics for the treatment of AIDS. These therapeutic RNA decoys and aptamers block HIV-1 transcription by perturbing the function of TAR. Some of them have been tested in clinical trials. As a continuation of his work on TAR, Teh identified a cellular TAR-binding protein named TRBP in 1991 by screening a cDNA expression library with a TAR RNA probe [3]. TRBP was later found to be a binding partner and regulator of double stranded RNA-dependent protein kinase PKR and Dicer protein that mediates RNA interference. Interestingly, a small molecule activator of TRBP was recently identified by others through a chemical screen. This compound named enoxacin can enhance TRBP-mediated microRNA processing and therefore exhibits cancer-specific and TRBP-dependent growth inhibitory effects [4]. Although through a circuitous route, Teh's identification of TRBP led to the development of a new agent that holds promise for the treatment of cancer.

Research on HTLV-1 that causes adult T-cell leukemia was another area close to Teh’s heart. He started his work on HTLV-1 when he was with George Khoury. Over the years he laid a number of cornerstones that shaped the field. His studies not only provided molecular details of the activation of viral and cellular transcription by HTLV-1 oncoprotein Tax mediated through CREB and NF-κB, but also derived mechanistic insights into Tax-induced oncogenic transformation of T lymphocytes. Existing and emerging evidence now supports his ideas that Tax creates genome instability by perturbing DNA damage response, mitotic checkpoint and centrosomal duplication [5]. Because frontline chemotherapeutic drugs for cancer target the DNA damage response or mitotic checkpoint, Teh’s findings had important therapeutic implications. In his George Khoury lecture delivered in October 2012, he summarized a part of his work on HTLV-1, which focuses on genome integrity and aneuploidy. Undoubtedly, Teh’s thoughts and findings will continue to have substantial and long-lasting impacts on HTLV-1 research.

While he made every effort to get to the bottom of the research questions he was asking, Teh always kept his curiosity alive and was ready to embrace serendipity. In his study of HIV-1 TAR he coincidentally identified TRBP [3], which is now known to be a key player in RNA interference. In his study of HTLV-1 Tax he identified MAD1 which is a core component of mitotic checkpoint [6]. In his study of MAD1 he inadvertently found that the absence of inner nuclear membrane protein SUN1 ameliorates laminopathy and premature aging [7]. All these serendipitous discoveries by Teh turned out to be path-breaking. They opened up avenues for new studies in several different fields.

In our personal views, in addition to his courage and wisdom, Teh’s success in science can also be attributed to three additional traits. First, he aimed high and worked extremely hard. One of our colleagues in Hong Kong received his email sent in the evening of Sunday, January 27, 2013, just hours before his death. In this email he made suggestions for manuscript preparation. He had been working essentially 7 days a week for 27 years, even when the NIH was shut down as a result of the budget battle or during snowstorms. No matter when and where we sent him an email, we got a reply immediately or within a few hours. Such work ethics that he inherited from the older generation of Chinese Americans was an important factor in his tremendous success. Second, he managed to make the best use of cutting-edge technologies to address important scientific questions. At least one new method was employed in each of his primary research papers cited above. For example, yeast two-hybrid technology was used in the identification of MAD1 [6], whereas double knockout mice were made to reveal the novel role of SUN1 in progeria [7]. He was always enthusiastic about new methodologies and technology development. He was actually one of the first in the fields of HIV-1 and HTLV-1 to do RNAi screening, deep sequencing and microRNA profiling. Finally, he constantly sought to make connections with talented and competent individuals. He was a serious and exemplary collaborator. Most of his papers cited above represent fruitful collaborations with others.

Teh had been very outspoken on issues affecting Asian American scientists, such as their representation in leadership positions. He joined the Society of Chinese Bioscientists in America (SCBA) as one of its earliest lifetime members. He was elected the President of SCBA for a two-year term from 2011–2012 [8]. During his tenure, Teh organized one of the most successful biennial meetings both scientifically and financially. He recruited a large number of new members both in North America and Asia. His tireless effort in promoting SCBA led to enormous donations to the society that helped to provide a solid financial foundation for future development. In addition, with the long-term view of a strong and successful society in mind, Teh envisioned the necessity and benefit of a society journal. This led him to create the official SCBA journal, *Cell and Bioscience*, in 2011 [9]. Teh was also instrumental in the tremendous success of this new journal, including its acceptance by Thomson Reuters for tracking, from the very first paper published*,* for an Impact Factor to be released later this year [10].

Teh was a superb mentor. He was attentive, supportive and at times demanding to his postdocs who came from all over the world. He generously gave personal and practical help to people in the lab on various issues. Importantly, for everything he asked from his postdocs, he set himself as a role model for them. Many postdocs became his lifelong friends. His commitments to his postdocs and friends were always strong. Teh told us stories about how his mentor George Khoury was caring and committed to his fellows. In this regard, they both were the type of supervisors and academic leaders who were not only outstanding scientists but also great human beings. Teh’s life was much too short, but his legacy and our memories of him will last forever. Our hearts and condolences are with his wife Diane and his three children David, Diana and John.
